# Vegfa Impacts Early Myocardium Development in Zebrafish

**DOI:** 10.3390/ijms18020444

**Published:** 2017-02-21

**Authors:** Diqi Zhu, Yabo Fang, Kun Gao, Jie Shen, Tao P. Zhong, Fen Li

**Affiliations:** 1 Department of Pediatric Cardiology, Shanghai Children’s Medical Center Affiliated to Shanghai Jiaotong University School of Medicine, Shanghai 200127, China; zhudiqi@scmc.com.cn (D.Z.); shenjie@scmc.com.cn (J.S.); 2 State Key Laboratory of Genetic Engineering, Zhongshan Hospital, School of Life Sciences, Fudan University, Shanghai 200438, China; yabofang@126.com (Y.F.); kungao2016@126.com (K.G.)

**Keywords:** Vegfa, cardiac fusion, zebrafish

## Abstract

Vascular endothelial growth factor A (Vegfa) signaling regulates cardiovascular development. However, the cellular mechanisms of Vegfa signaling in early cardiogenesis remain poorly understood. The present study aimed to understand the differential functions and mechanisms of Vegfa signaling in cardiac development. A loss-of-function approach was utilized to study the effect of Vegfa signaling in cardiogenesis. Both morphants and mutants for *vegfaa* display defects in cardiac looping and chamber formation, especially the ventricle. Vegfa regulates the heart morphogenesis in a dose-dependent manner. Furthermore, the initial fusion of the bilateral myocardium population is delayed rather than endocardium. The results demonstrate that Vegfa signaling plays a direct impact on myocardium fusion, indicating that it is the initial cause of the heart defects. The heart morphogenesis is regulated by Vegfa in a dose-dependent manner, and later endocardium defects may be secondary to impaired myocardium–endocardium crosstalk.

## 1. Introduction

Congenital heart diseases (CHD) occur when genetic and/or environmental disturbances undermine normal cardiac development [1]. Fully understanding the genetic pathways and their interactions that influence cardiogenesis may help explain the mutations found in CHD patients [2]. Fate-mapping studies have demonstrated that the heart forming region lies within the anterior lateral plate mesoderm (ALPM) [3]. The bilateral regions of ALPM will migrate towards the midline to form a primitive linear heart tube [4]. The heart tube is composed of an outer myocardial and an inner endocardial cell layer that will give rise to the cardiac valves and septa in later development.

Several studies have proven that vascular endothelial growth factor A (Vegfa) plays a crucial role in vascular development; however, a detailed examination of its function in cardiogenesis has not yet been conducted. The heart region in heterozygous VEGF-deficient mice (*VEGF*^+/−^) displayed atrium and ventricle developmental delay and decreased thickness of the ventricle wall [5]. *VEGF*^+/−^ is embryonically lethal due to severe vascular anomalies that lose the chance to generate homozygous-deficiency model for in vivo study [5,6].

Several studies clue that Vegfa signaling may be required in the late stage of heart development: the formation of cardiac valves [7,8]. The absence of the Vegf_164_ isoform in mouse results in the malformation of interventricular septum and septation of the cardiac outflow tract [9]. VEGF receptor deficiency by treated with selective tyrosine kinase inhibitors resulted in a functional and structural heart valve defect and loss of cell-restricted expression *notch1b* and bone morphogenetic protein 4 (*bmp4*) at the atrio-ventricular canal (AVC) in zebrafish embryos [10]. Previous studies show that VEGF-induced nuclear factor of activated T-cells, cytoplasmic, calcineurin-dependent 1 (*NFATc1*) activation may promote endocardial cell proliferation for maintaining endocardial cell numbers in heart valve development [11,12], but we have limited knowledge about the VEGF-mediated regulation of cardiogenesis before the formation of AVC. Whether Vegfa influences the myocardium or endocardium is yet uncertain. Therefore, to address the role of Vegfa signaling in cardiac development, further studies are essential.

In this study, we find that Vegfa signaling plays a major role in cardiac development in the zebrafish embryo. Using loss-of-function approaches, we demonstrate that Vegfa signaling is essential for cardiomyocytes fusion rather than endocardium. Genetic ablation of Vegfa signaling results in flaws during cardiac looping and chamber formation. Vegfa promotes cardiomyocytes proliferation, especially for the ventricle. Together, these results reveal that Vegfa signaling exerts an early and direct impact on cardiac morphogenesis.

## 2. Results

### 2.1. Vegfaa Deficiency Disrupts Myocardial and Endocardial Morphogenesis

**Figure 1:**
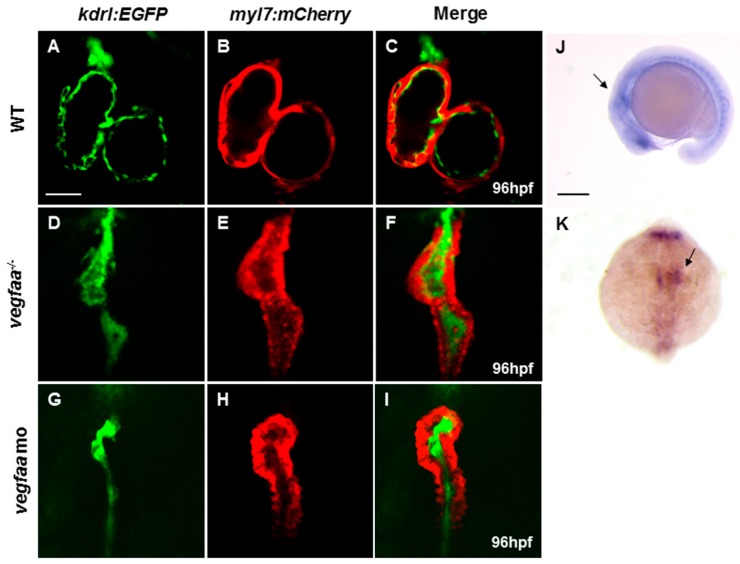
Vascular endothelial growth factor Aa (*vegfaa*) deficiency disrupts myocardial and endocardial morphogenesis. (**A**–**I**) Fluorescence micrographs of the hearts of wild-type (**A**–**C**), *vegfaa* mutant (**D**–**F**), and *vegfaa* morphants (**G**–**I**) at 96 hpf (hours post-fertilization) with the background of *Tg*(*myl7:mCherry*)/*Tg*(*kdrl:EGFP*) transgenic fish. The whole heart is substantially small and linearized in the mutant and the morphant, and no cavity is visible inside the heart lumen. Scale bar: 50 μm (**A**–**I**); (**J**,**K**) In situ hybridization of *vegfaa* in wild-type embryos at 18-somite stage. *vegfaa* is expressed in anterior lateral plate mesoderm as indicated by the arrow. (**J**) Lateral views, (**K**) Dorsal views with the anterior at the top. Scale bar: 200 μm (**J**,**K**). WT, wild-type; mo, morpholino.

Zebrafish harbor duplicate copies of *vegfa* genes homologous to Vegfa in human and mouse, known as *vegfaa* and *vegfab* [13]. First, we examined the expression of *vegfaa* and *vegfab*, respectively. *vegfaa* is expressed at mesoderm underlining and lateral to the posterior hindbrain, which is in or in proximity to the prospective heart fields at 18-somite stage (Figure 1J,K), in agreement with the previous reports [14]. Expression of *vegfaa* in the heart field was downregulated after early migration. *vegfab* expression was not detected during early embryonic development (Figure S1C,D). We also used antisense morpholino oligonucleotides (MO) that targeted the *vegfab* splicing site, which effectively reduced the normal *vegfab* transcription levels (Figure S1A,B). The gross morphology and heart development were almost normal with 10 ng doses of *vegfab* MO (Figure S1E). Therefore, we speculated that *vegfaa* dominantly functions in Vegfa singling. For *vegfaa*, we used splicing blocking MOs, which retains the intron sequences in the messenger RNA (mRNA) and successfully downregulated the *vegfaa* transcription levels (Figure S2A,B).

Furthermore, we analyzed the *vegfaa* mutants generated using the TALEN (transcription activator-like (TAL) effector nuclease) genome editing approach. The *vegfaa* allele, thus obtained, harbors a 7 bp deletion resulting in a premature stop codon at position 18, representing a null allele [15]. Bright-field microscopy analysis revealed loss of circulation, pericardial edema at 48 and 72 hpf (hours post-fertilization), and death at 5-days post-fertilization (dpf) in *vegfaa* mutants, which is consistent with *vegfaa* morphants reported previously [16] and the current morphants. Our *vegfaa* morphants and mutants display identical vascular defects in arterial-venous differentiation by whole-mount in situ hybridizations (WISH) analysis of cadherin 5 (*cdh5*), an endothelial marker (Figure S2C). These data demonstrate that the current *vegfaa* morphants and mutants models are reliable and exclude the off-target possibility.

In addition to defects in vascular development, we observed a strong defect in the morphogenesis of the heart in *vegfaa* mutants and morphants. In order to further investigate the cardiac morphogenesis, we analyzed the *vegfaa* mutant in the backgrounds of two transgenic fish lines: *Tg*(*myl7:mCherry*) that labels the myocardium, and *Tg*(*kdrl:EGFP*) that labels the endocardium. As observed using these transgenic lines, both the ventricle and atrium in the mutants were markedly shrunken, and the heart failed to loop normally compared to the wild-type siblings (Figure 1A–F). The mutant heart appeared compact and tubular. Although the initial heartbeat was normal and the endocardial cells could be distributed in both ventricle and atrium, the endocardium cells almost filled with the heart capacity leading to nearly no valid lumen or heart valve.

### 2.2. Vegfaa Regulates Heart Development in a Strict Dose-Dependent Manner

**Figure 2:**
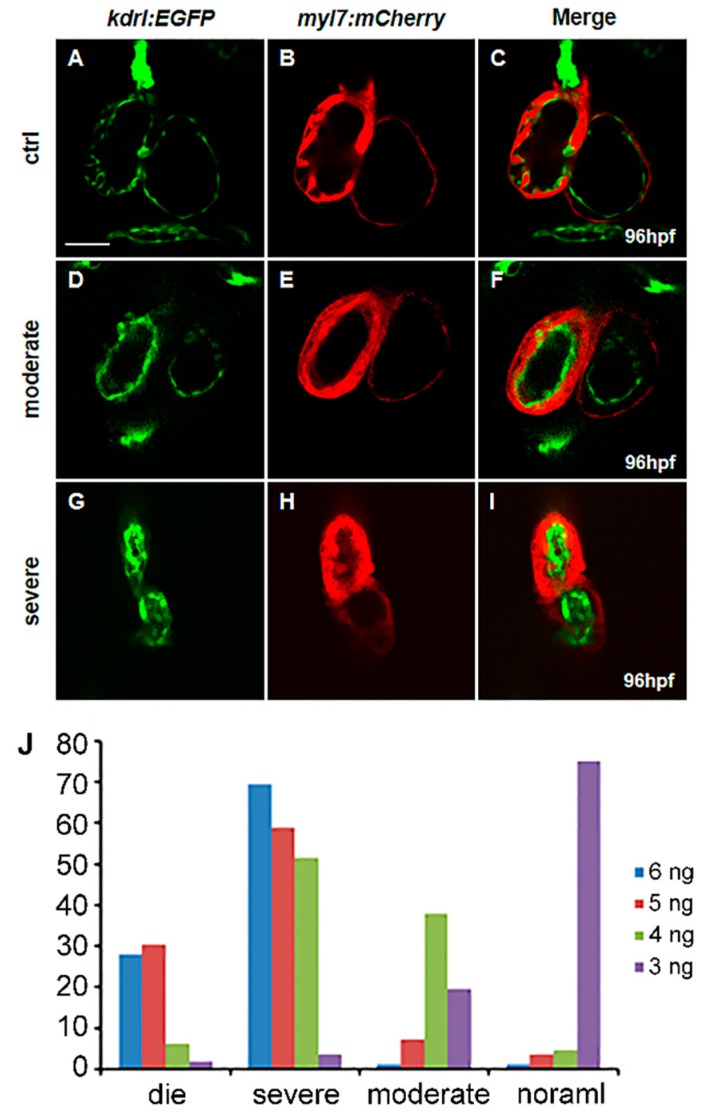
*vegfaa* regulates heart development in a dose-dependent manner. (**A**–**I**) Fluorescence micrographs of the hearts of wild-type (**A**–**C**), *vegfaa* morphants with moderate defects (**D**–**F**), and severe defects (**G**–**I**) at 96 hpf in the background of *Tg*(*myl7:mCherry*)*/Tg*(*kdrl:EGFP*) transgenic fish, Scale bar: 50 μm; (**J**) Statistical analysis of MO dosage and heart malformation in *vegfaa* morphants.

*vegfaa* morphants also display cardiovascular defects but are variable with different MO doses (Table S1). We define “moderate defects” as the heart being normally or almost normally looped, and the myocardium and endocardium are well formed. These two layers cling to each other, and the heart has the valid capacity. The endocardial cells are accumulated at AVC forming heart valve and some of myocardial cells are projected to the ventricle capacity called trabeculation. However, the “moderate defects” may show defects in valve formation and trabeculation (Figure 2D–F). “Severe defects” are defined as the shrunken heart and linearized identical to the *vegfaa* mutants above (Figure 2G–I).

The majority of the embryos display normal heart development (42/56) or moderate defects (11/56) with 3 ng MO doses. With a slight increase in the dose of MO to 4 ng, approximately half of the morphants show severe defects (34/66) and a proportion with moderate malformation (25/66). The heart phenotypes are severe as the dose is increased up to 5 ng MOs (17/56 embryonic death, and 33/56 severe defects), while most of embryos are dead (23/82) or display severe defects (57/82) when 6 ng *vegfaa* MOs is administered. These results show that cardiac development in zebrafish is extremely sensitive to Vegfa dose and in a stringent dose-dependent manner.

### 2.3. Early Myocardial/Endocardial Development in the Vegfaa Mutant

Since cardiac function was impaired in *vegfaa* mutants at late developmental time points, we examined whether these late defects arise from an earlier requirement for Vegfa signaling. To assess the initial cardiac progenitors emergence, we tested the markers of cardiac precursors, the transcription factor NK2 homeobox 5 (*nkx2.5*) [17] and ALPM marker heart and neural crest derivatives expressed 2 (*hand2*) [18] by WISH analysis. Both were unaffected in the *vegfaa* mutants (Figure S3A–D), indicating that the initial establishment of the bilateral ALPM, as well as cardiac progenitors, occurs appropriately despite the loss of *vegfaa* gene function. Next, we examined *myl7*-expressing differentiated cardiomyocytes at 17-somite stage. The unaltered number and intensity of *myl7* expression reveal normal differentiation of bilateral cardiac precursor populations (Figure S3E,F).

**Figure 3:**
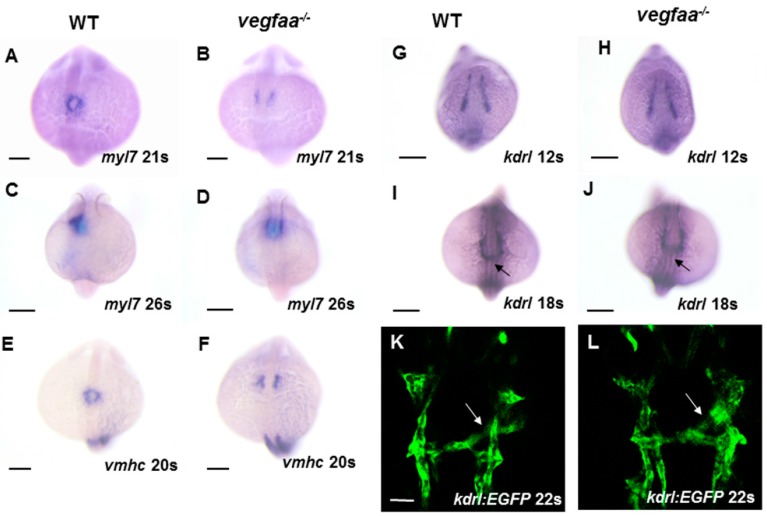
*vegfaa* mutants display defects in cardiomyocytes fusion. (**A**–**D**) In situ hybridization depicts *myl7* expression at 21- and 26-somite stage in wild-type embryos (**A**,**C**) and *vegfaa* mutant embryos (**B**,**D**). At 21-somite stage, the wild-type cardiomyocytes formed a ring (**A**), whereas few cardiomyocytes are formed in the *vegfaa* mutants (**B**). At 26-somite stage, the wild-type primitive heart tube begins left looping (**C**) while *vegfaa* mutant embryos are still cardiac cone (**D**); (**E**,**F**) In situ hybridization depicts ventricular myosin heavy chain (*vmhc*) expression at 20-somite stage in wild-type embryos (**E**) and *vegfaa* mutant embryos (**F**); (**G**–**J**) Dorsal views depict unaffected endocardial expression pattern of *kdrl* in *vegfaa* mutants. Black arrows indicate midline migration of endocardial cells; Scale bar: 200 μm (**A**–**J**); (**K**,**L**) Dorsal views depict endocardial expression of *Tg*(*kdrl:EGFP*) at the 22-somite stage. White arrows indicate leftward movement. Scale bar: 50 μm.

**Figure 4:**
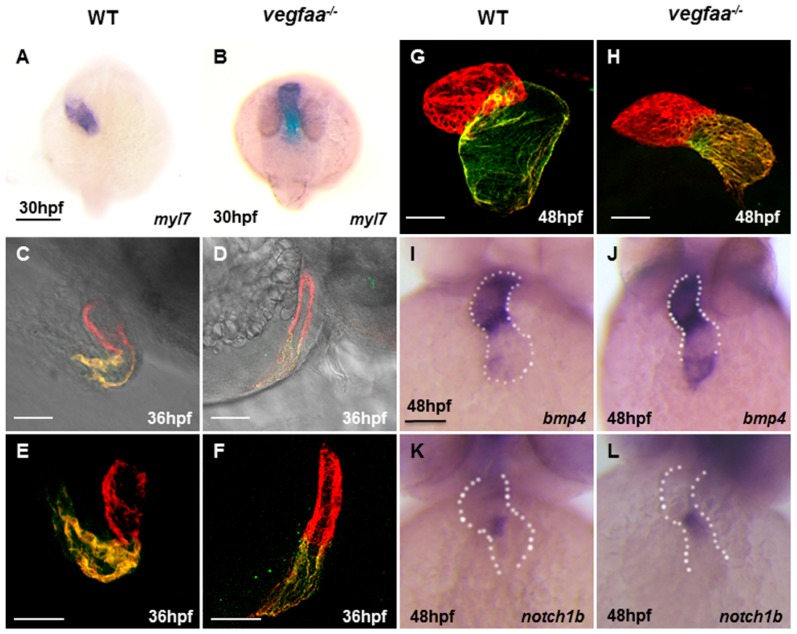
*vegfaa* deficiency results in defects in cardiac looping and chamber formation. (**A**,**B**) Dorsal views of in situ hybridization depicts *myl7* expression at 30 hpf in wild-type embryos (**A**) and *vegfaa* mutant embryos (**B**). Scale bar: 200 μm (**A**,**B**); (**C**–**F**) Lateral views of zebrafish hearts stained with MF20 (red) and S46 (green) antibodies to visualize the ventricle and atrium at 36 hpf. MF20 marks the whole heart, whereas S46 is atrium-specific. Scale bar: 50 μm. (**C**,**D**) are added with light microscopy; (**G**–**H**) At 48 hpf, the wild-type heart is looped, with morphologically distinct chambers, whereas the *vegfaa* mutant heart appears unlooped, with a small heart, especially ventricle. Scale bar: 50 μm (**C**–**H**); (**I**–**L**) Frontal views depict expression of the atrio-ventricular canal (AVC) markers in wild-type (**I**,**K**) and in *vegfaa* mutants (**J**,**L**) at 48 hpf. Dotted lines outline the chambers flanking the AVC. Scale bar: 100 μm (**I**–**L**).

**Figure 5:**
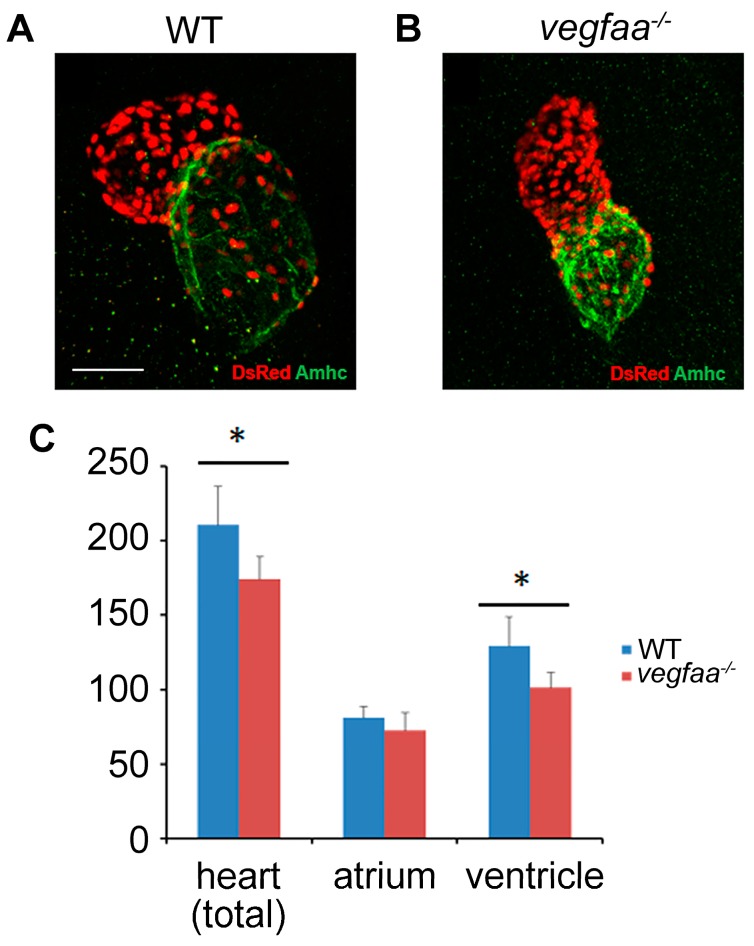
*vegfaa* promotes cardiomyocytes proliferation. (**A**,**B**) Immunofluorescence indicates the expression of transgene *Tg*(*myl7:nDsRed*) (red) in both cardiac chambers facilitating cardiomyocyte counting at 48 hpf. Atria are labeled with the anti-Amhc (atrial myosin heavy chain) antibody S46 (green); (**C**) Bar graphs indicate the number of atrial and ventricular cardiomyocyte nuclei, as well as the total number of cardiomyocytes; asterisks indicate statistically significant differences compared to wild-type (*p* < 0.05) (WT *n* = 5; *vegfaa^−/−^* *n* = 5).

However, the cardiac fusion delay and abnormal heart tube looping become apparent in *vegfaa* mutants during cardiac fusion later (Figure 3A–D). In wild-type embryos, the cardiomyocytes migrate to midline and form a cardiac ring at 21-somite stage (Figure 3A). The medial migration of cardiomyocytes in *vegfaa* mutants was significantly stalled (Figure 3B). However, the bilateral populations of cardiomyocytes were not permanently separated, and eventually shaped the cone at 26-somite stage (Figure 3D), forming two linked sets of dysmorphic chambers. After assessing the *myl7*-expression, we also analyzed the ventricular myosin heavy chain (*vmhc*) expression at 20-somite stage, and our data indicated that ventricle responds with increased sensitivity to Vegfa signaling (Figure 4 and Figure 5). In agreement with our prior results, loss of Vegfa signaling inhibited *vhmc*-expressing ventricular cardiomyocytes migration to midline (Figure 3E,F); however, the *vmhc* intensity seems unchanged. These results demonstrate that the defects during cardiomyocytes fusion rather than generation and differentiation are the initial cause of the heart defects observed in *vegfaa* mutant embryos.

Since *vegfaa* is expressed at ALPM at 18-somite stage and can be diffused in proximity, we tested whether Vegfa signaling is also required for endocardium formation; *kdrl* expression was analyzed during the early somitogenesis stages in *vegfaa* mutants. In the wild-type embryos, *kdrl* is expressed in all of the endothelial cells including the endocardium, but not the myocardium, of the primitive heart tube. Any significant defects were not observed in the endocardial marker expression in *vegfaa* mutants compared to the wild-type siblings during the endocardial precursors origination, migration to midline, and leftward movement (Figure 3G–L). These data suggest that the early formation of endocardial cells is independent of Vegfa signaling.

### 2.4. Vegfaa Deficiency Disrupts Cardiac Looping and Chamber Formation

After cardiac fusion, the heart tube will initiate leftward looping and extension. *vegfaa* mutant embryos exhibit improperly leftward-looped hearts and the heart tube extends aberrantly and develops a narrow but longer heart tube (Figure 4A–F). The heart is maintained in the middle, exhibiting a small, narrowed heart chamber, especially the ventricle at 48 hpf (Figure 4G,H). In wild-type embryos, the markers associated with endocardial differentiation, such as *bmp4* and *notch1b*, are expressed throughout the endocardium at 24 hpf and later enriched in the atrio-ventricular canal (AVC) at 48 hpf [19,20]. In *vegfaa* mutants, *bmp4* expression was expanded into the ventricular myocardium while *notch1b* remained unaltered (Figure 4I–L). The intensity of these two unchanged genes indicated almost normal endocardium differentiation but defects in valve morphogenesis. The ectopic expression of bmp4 in *vegfa* deficiency embryos may be due to Vegfa-bmp/TGFβ signaling interaction.

### 2.5. Vegfaa Promotes Cardiomyocytes Proliferation

The cardiomyocytes in wild-type and *vegfaa* mutant embryos were counted in order to evaluate whether the number of cells in each chamber were synchronously decreased or increased as a destiny for cardiomyocytes. At 48 hpf, we observed a reduction in ventricular cells as well as the total number of cardiomyocytes, and no statistically significant difference in atrial cardiomyocytes (Figure 5A–C). The number of ventricular cardiomyocytes was more affected than the number of atrial cardiomyocytes. Altogether, the predisposition of reduced cardiac cell number in embryos with disrupted Vegfa signaling demonstrates a role for Vegfa in promoting cardiomyocyte proliferation.

## 3. Discussion

Using genetic loss-of-function approaches, we found that Vegfa signaling is required for myocardium migration rather than endocardium. *vegfaa* mutant embryos first develop subtle morphological aberrations from cardiac migration to midline, foreshadowing the later phenotype defects in the heart tube looping and chamber formation. The cardiogenesis in zebrafish embryos is regulated by stringent dose-dependent control of Vegfa signaling.

Previous studies showed that the early cardiac phenotype of silent heart (*sih^−/−^*) embryos, which establish neither a heartbeat nor blood flow due to a mutation in cardiac troponin T (*tnnt2*), undergo normal looping morphogenesis and chamber differentiation [21]. These results support that early cardiac morphogenesis is independent of circulation and exclude the possibilities that *vegfaa* mutants are secondary to vascular defects. Therefore, we proposed that the Vegfa signaling has a direct impact on cardiac morphogenesis.

In the current study, we found that *vegfaa* mutants display defects in cardiomyocytes fusion rather than endocardial cells. Vegfa morphants showed delayed heart field fusion for both endocardial and myocardial cells as reported previously [22]. Another study argued that Vegfa MO knockdown did not show any defects in endocardial marker expression [20]. These contrasting observations might be attributable to Vegfa MO knockdown that cannot fully eliminate the Vegf function. Our genetic mutation approaches prove that Vegfa is essential for myocardial migration but is dispensable for endocardial morphogenesis. This phenotype is consistent with the hypomorphic Vegfa knock-in allele, which does not show apparent defects in endocardial morphogenesis in the mouse model [23].

Endocardial and myocardial cells closely interact during development. We discovered endocardium distribution defects and loss of valve formation at a later stage despite the normal generation, migration, and differentiation of endocardial cells. Thus, this phenomenon may be ascribed to endocardium–myocardium crosstalk. Previous studies showed that endocardial cells are crucial for the migration of myocardial progenitors during cardiac cone assembly and myocardial trabeculation [24,25]. On the other hand, the myocardium is vital for endocardial morphogenesis and differentiation [26]. Since myocardium migration is impeded in *vegfaa* mutants and displays compact chamber of myocardium layer, endocardium malformation may be secondary to damaged myocardium function.

## 4. Materials and Methods

### 4.1. Zebrafish Lines and Maintenance

All the fish used for the experiments including wild-type (AB), mutants, and transgenics were maintained at 28.5 °C. The transgenic lines used in this study include *Tg*(*kdrl:EGFP*) [27], *Tg*(*myl7:mCherry*) [28], and *Tg*(*myl7:nDsRed*) [29]. To maintain optical clearance, the embryos were treated with 0.003% phenylthiourea (PTU) to suppress pigmentation for analysis performed beyond 24 h post-fertilization (hpf).

### 4.2. Morpholino Knockdown

All morpholinos (MO) were procured from GeneTools and injected at the specified doses into 1- to 2-cell stage embryos. The sequences for *vegfaa* translation blocking morpholino: 5′-GCTGGATTAAAGCTGTCTCACCTCC-3′ and *vegfab* translation blocking morpholino: 5′-TGGAAGTAAGGAGTCCCTGACCTCC-3′. Primers used for RT-PCR were listed in Table S2.

### 4.3. In Situ Hybridization and Immunohistochemistry

Whole-mount in situ hybridizations (WISH) were performed as described previously [30]. Digoxigenin-labeled probes for *vegfaa*, *vegfab*, *kdrl*, *myl7*, *amhc*, *vmhc*, *nkx2.5* and *hand2* were transcribed using T7 and SP6 RNA polymerase (Invitrogen, Carlsbad, CA, USA).

Whole-mount immunofluorescence was performed as described previously [30], using primary monoclonal antibodies against sarcomeric myosin heavy chain (MF20) and atrial myosin heavy chain (S46). MF20 and S46 were obtained from the Developmental Studies Hybridoma Bank (DSHB, Iowa City, IA, USA). The secondary antibodies used were goat anti-mouse IgG1 FITC and goat anti-mouse IgG2b TRITC (both 1:100; Southern Biotechnology Associates, Birmingham, AL, USA).

### 4.4. Cardiomyocyte Counting

To count the cardiomyocytes, we processed *Tg*(*myl7:nDsRed*) transgenic embryos using a standard immunofluorescence protocol [31]. The primary antibodies used were S46 (anti-Amhc; 1:20; DSHB) and anti-DsRed (1:4000; Clontech, Mountain View, CA, USA), and the corresponding secondary antibodies were goat anti-mouse IgG1 FITC (1:100; Southern Biotechnology Associates) and donkey anti-rabbit Alexa 594 (1:1000; Invitrogen). The red fluorescent nuclei in each cardiac chamber were enumerated.

### 4.5. Imaging

Transgenic and stained embryos were embedded in low melting temperature agarose, and images were acquired using Zeiss LSM 710 confocal microscope (Carl Zeiss, Jena, Germany). The acquired *Z*-stacks were processed using Imaris software (Imaris 7.6, Biteplane, Zurich, Switzerland).

### 4.6. Statistical Analysis

The statistical analysis was performed by Student’s *t*-test and Chi square test using the SPSS19.0 software (SPSS 19.0, IBM, Armonk, NY, USA). A *p*-value of less than 0.05 was considered significant in all cases (* *p* < 0.05; ** *p* < 0.01; *** *p* < 0.001).

## Supplementary Materials

Supplementary materials can be found at www.mdpi.com/1422-0067/18/2/444/s1.

## Abbreviations

CHD	Congenital heart diseases
ALPM	Anterior lateral plate mesoderm
AVC	Atrio-ventricular canal
MO	Morpholino oligonucleotides
TALEN	Transcription activator-like (TAL) effector nuclease
WISH	Whole-mount in situ hybridizations
DSHB	Developmental Studies Hybridoma Bank
