# Supplementary material for: Vegfa Impacts Early Myocardium Development in Zebrafish
Source: Int J Mol Sci. 2017 Feb 21;18(2):444. doi: 10.3390/ijms18020444 (PMC5343978; doi:10.3390/ijms18020444)
Supplement: Supplementary file 1 [file ijms-18-00444-s001.pdf]

Supplementary Materials: Vegfa Impacts Early Myocardium Development in Zebrafish

Diqi Zhu, Yabo Fang, Kun Gao, Jie Shen, Tao P. Zhong and Fen Li

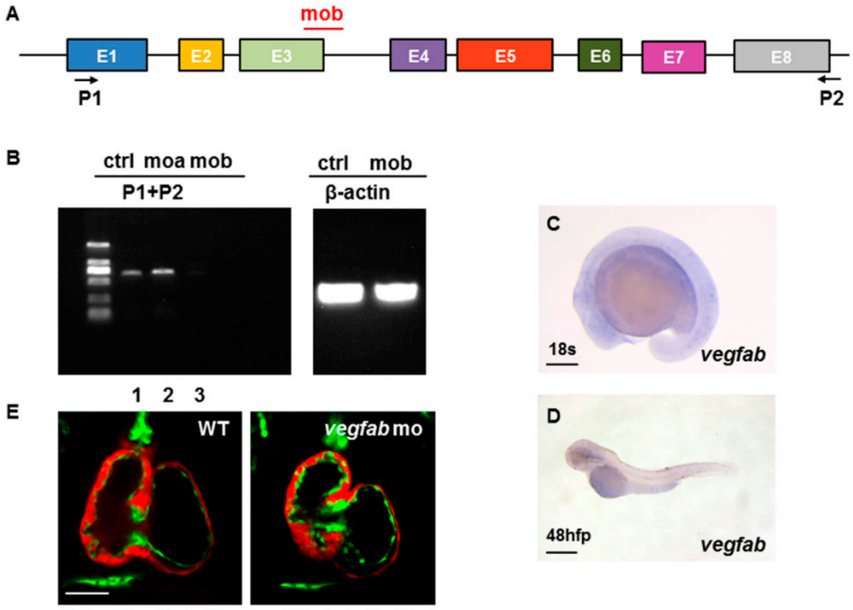

Figure S1. *vegfab* is not involved in early cardiovascular development in zebrafish. (**A**) Open boxes and black lines denote exons and introns of the early coding and non-coding region of *vegfab*. MO in red mark the target sites of the *vegfab* translation blocking. P1 and P2 indicate the sites of primers; (**B**) Fragments of *vegfab* sequences were amplified using the indicated primers. In *vegfab* morphant embryos, the normal *vegfab* RT-PCR product was not detected (lane 3). *β-actin* cDNA was also amplified from each sample as an internal control; (**C**,**D**) The expression of *vegfab* during early embryogenesis cannot be detected. Scale bar: 200 μm (**C**); 500 μm (**D**); (**E**) The heart development is almost normal with 10ng doses of *vegfab* MO at 96 hpf under the background of *Tg*(*myl7:mCherry*)/*Tg*(*kdrl:EGFP*) transgenic fish. Scale bar: 50 μm.

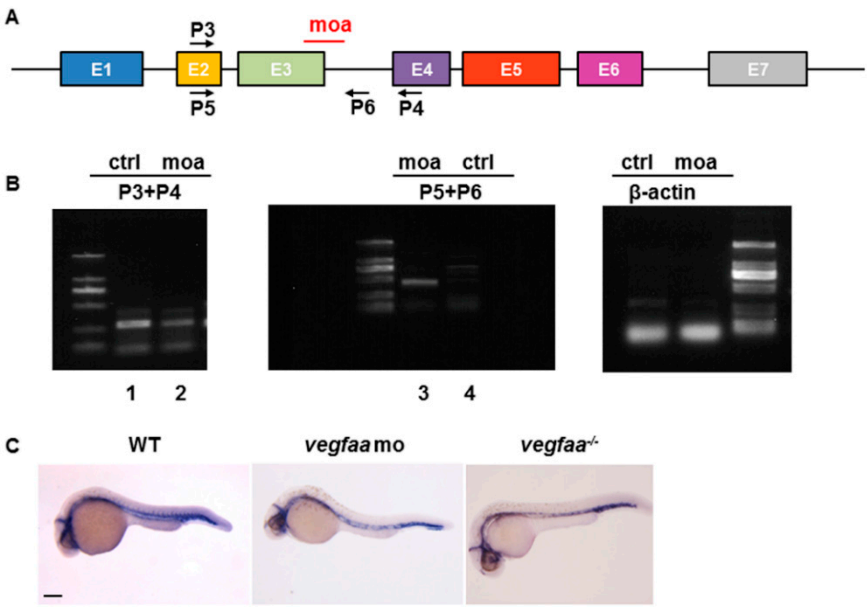

Figure S2. RT-PCR validation of morpholino efficacy for *vegfaa*. (**A**) Open boxes and black lines denote exons and introns of the first coding and non-coding region of *vegfaa*. MO in red mark the target sites of the vegfaa translation blocking. P3, P4, P5, and P6 indicate the sites of primers; (**B**) One- to two-cell embryos were injected with *vegfaa* MO, and cDNA at 24 hpf was isolated. In wild-type embryos, the normal *vegfaa* spliced P3–P4 RT-PCR product was observed (lane 1) but not the P5–P6 splice donor site PCR product (lane 3). In *vegfaa* morphants, the normal transcrpiction level is decreased (lane 2) and the intron sequences are retained (lane 3). The *β-actin* cDNA was also amplified from each sample as a reference control; (**C**) Whole-mount in situ hybridizations (WISH) analysis of *cdh5* in WT, *vegfaa* morphants, and mutants at 30 hpf. The morphants and mutants display identical phenotype in arterial-venous differentiation and intersegmental vessel sprouting. Scale bar: 200 μm.

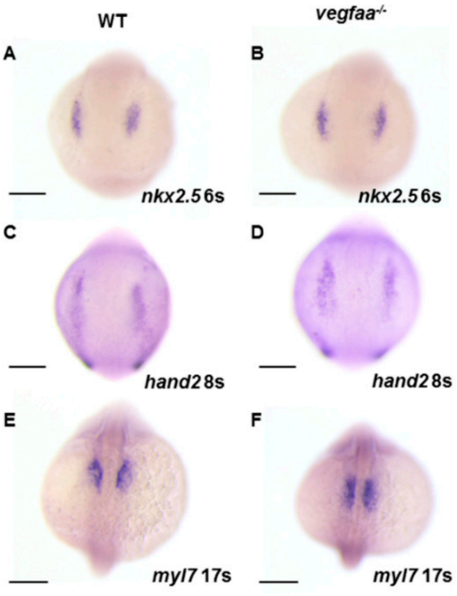

Figure S3. Normal pattern of cardiac progenitors and differentiation in *vegfaa* mutant embryos. (**A**–**D**) WISH demonstrates the expression of *nkx2.5* (**A**,**B**) and *hand2* (**C**,**D**) in wild-type (**A**,**C**) and *vegfaa* mutant (**B**,**D**) embryos; (**E**,**F**) WISH demonstrates the expression of *myl7* in wild-type (**E**) and *vegfaa* mutant (**F**) embryos at 17-somite stage. Dorsal views anterior to the top. Gene expression patterns appear normal in *vegfaa* mutants; no expansion or reduction of heart field size is evident. Scale bar: 200 μm (**A**–**F**).

Table S1. Effect of *vegfaa* morpholino dosage on cardiac morphogenesis.

MO Dosage	Die	Severe	Moderate	Noraml	Total
6 ng	23	57	1	1	82
5 ng	17	33	4	2	56
4 ng	4	34	25	3	66
3 ng	1	2	11	42	56

Table S2. List of primers used for RT-PCR.

Primer	5′—3′
P1	GCTTTCTGAGACCTTTACCAATGC
P2	TCTCAAGTCAGTTCGGTTCACCTCC
P3	CCAAAGAAGGGGGAAAGAGCA
P4	ATTATGCTGCGATACGCGTTG
P5	CCCACATACCCAAAGAA
P6	GCTGTCATCATGGCAAA
